# 

**Published:** 1915-10-02

**Authors:** 


### CONTENTS.

PAGE

**Doctors and the War** ... ... ... ... ... 1

**The Medical Profession and the New Taxes** ... 2

**Hospital and Institutional News** ... ... ... 3

A Gift to Dewsbury Infirmary—Censoring the German Medical Press—No Room for Slackers—The Problem of the Insured Tuberculous Patient—Long Voyages and the Food Supply—The Consumptive Recruit—The Closing of Poor-Law Institutions—The Control of Measles—A Pleasing Incident at King George Hospital—Cholera in the German Army—Potions for Pale Proletarians—A Curious Advertisement—Failures in the Profession—Army Medical Service Organisation—How Doctors are Wasted—Medical Defence in War Time—Panel Doctors' Worries—Evening Dinners for School Children—Is Cancer Contagious?—Factories for Consumptive Shoemakers—Will Free Choice of Doctor have to Go?—"An Unfair Burden"—Industrial Fatigue and its Consequences—A Hint for the King's Fund—This Week's Drug Market.

**A Clinical Pilgrimage in War Time** ... ... 9

The Children's Hospitals of Europe.

**How the Germans Treat Wounded Prisoners** 10

The Testimony of a French Doctor.

**The Royal Army Medical Corps** ... ... ... 11

The Distribution of Medical Officers in the Expeditionary Force.

PAGE

**Resources of Voluntary Hospitals**... ... ... 13

Some Comparisons Between London and Liverpool.

The Living and Dead Hands.

**Round the World** ... ... ... ... ... 14

Fellow-Workers and the Roll of Honour.

**The Heating of Hospitals** ... ... ... ... 15

III. The Royal Hospital for Sick Children, Glasgow.

**Buildings Contemplated** ... ... ... ... 16

**The Institutional Library** ... ... ... ... 17

The Military Surgery of To-day.

**Parliament and the Profession** ... ... ... 19

The Current Week's Proceedings.

**Passing Topics of the Day** ... ... ... ... 20

**The Editor's Letter-Box** ... ... ... ... 20

Our Limbless Sailors and Soldiers: An Appeal.

**The Institutional Worker** ... ... (Suppl.) 1

Hospital Administration.
Question for October.
Questions Invited.
Enquiries and Answers.
War Matters.
Miscellaneous.
The Housekeeper's Department.
Employment and Training.

